# Supplementary figures and images for: Infection of human organoids supports an intestinal niche for Chlamydia trachomatis
Source: PLoS Pathog. 2024 Aug 22;20(8):e1012144. doi: 10.1371/journal.ppat.1012144 (PMC11340892; doi:10.1371/journal.ppat.1012144)

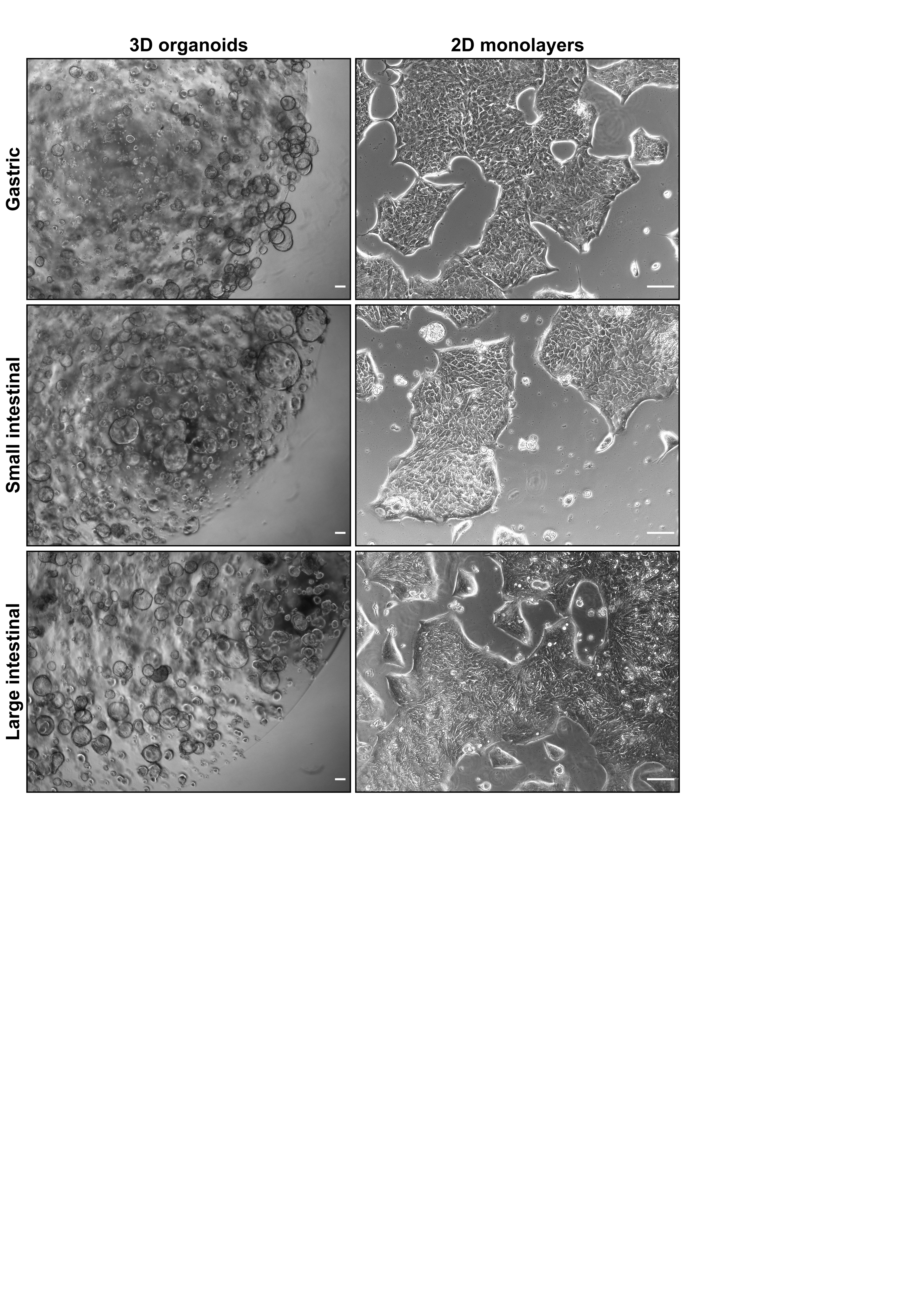

Supplement: S1 Fig — Representative phase-contrast images of the human gastric (corporal), small intestinal (jejunal), large intestinal (colonic) organoids in a Matrigel drop and the subconfluent 2D monolayers derived from respective organoid cultures in microwell plates. Scale bar: 100 μm. (TIF) [file ppat.1012144.s001.tif]

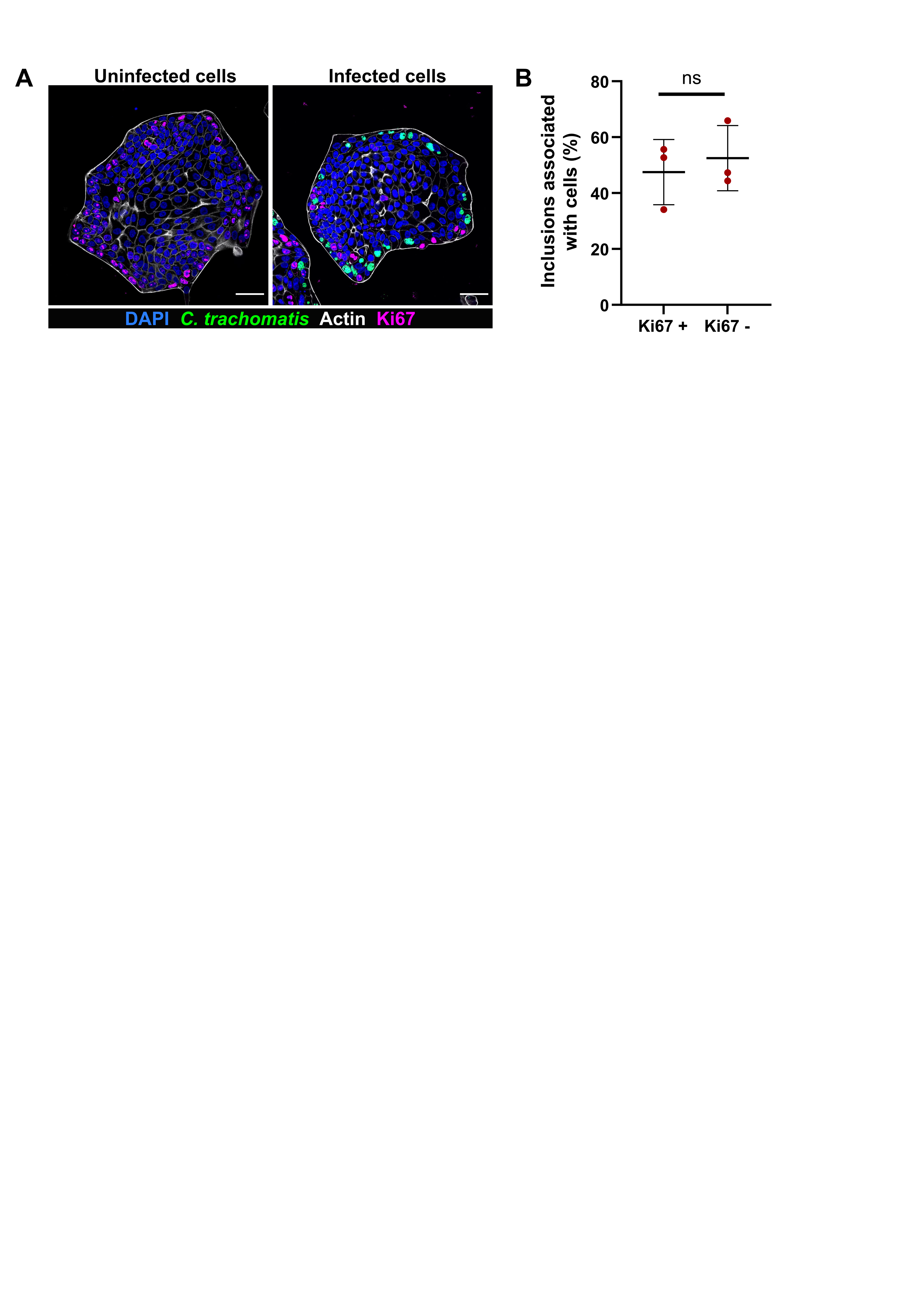

Supplement: S2 Fig — (A) Subconfluent gastric cells in microwell plates were infected with GFP-expressing C. trachomatis (MOI of 5) and 24 hours p.i. fixed, stained and subjected to confocal microscopy. Representative fluorescence microscopic images of three independent experiments show the localization of Ki67-positive cells in uninfected and infected samples (blue: DAPI, green: C. trachomatis, grey: actin, magenta: Ki67). Scale bar: 50 μm. (B) The percentage of the chlamydial inclusions residing in Ki67-positive or negative cells was determined by manually quantifying inclusions in five fields of view per sample. Data represent the mean ± SD from three independent experiments. Statistical analysis was performed by unpaired t-test (ns = not significant). (TIF) [file ppat.1012144.s002.tif]

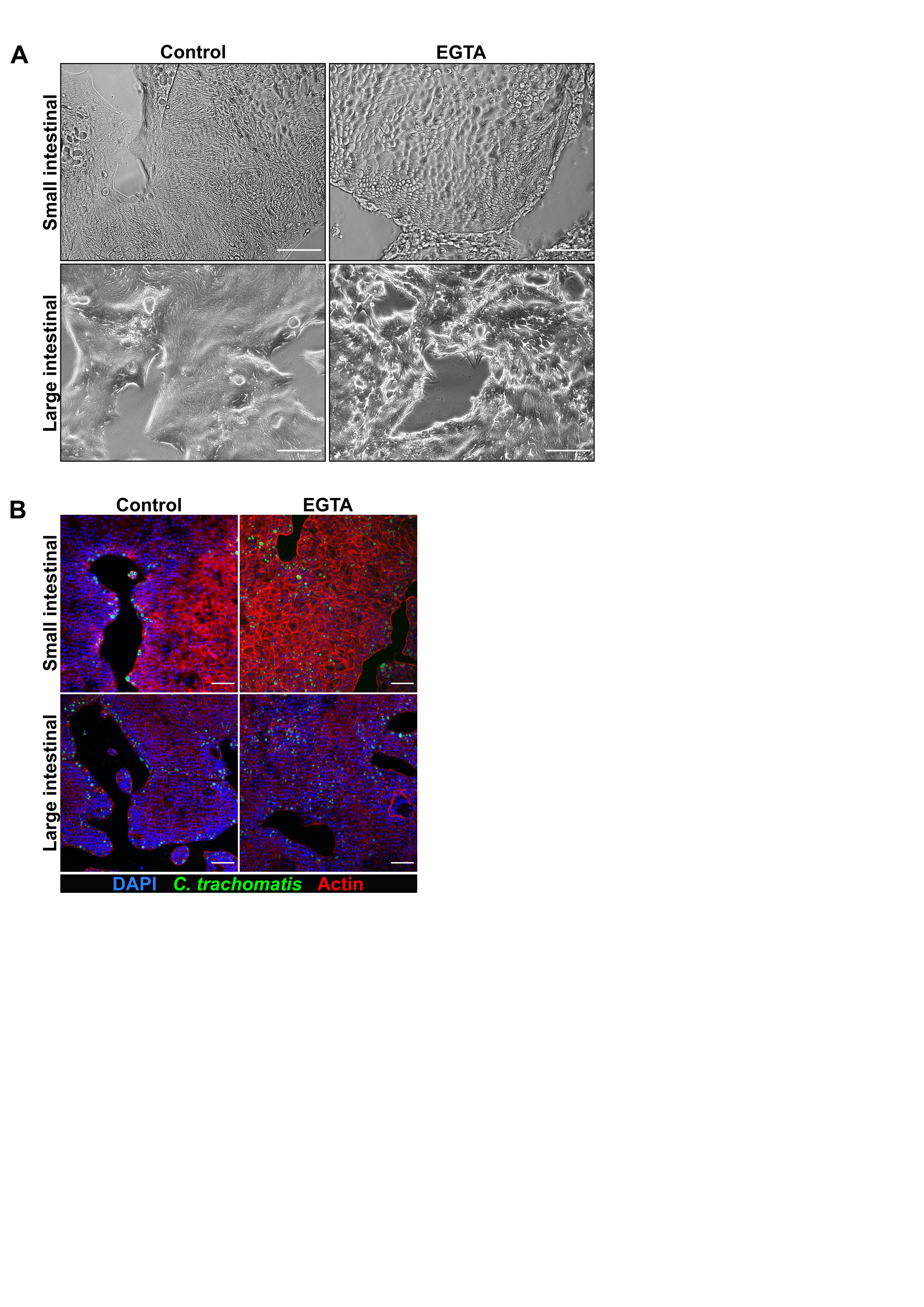

Supplement: S3 Fig — (A) Small and large intestinal epithelial cells grown as subconfluent monolayers were treated with 4 mM EGTA for 30 min at 37°C or left untreated. Phase-contrast images show the changes in the morphology of the cells upon treatment. Scale bar: 100 μm. (B) Intestinal cells pre-treated with 4 mM EGTA for 30 min or left untreated, were infected with GFP-expressing C. trachomatis (MOI of 5). 24 hours p.i. the cells were fixed, stained and subjected to confocal microscopy (blue: DAPI, green: C. trachomatis, red: Actin). Scale bar: 100 μm. (TIF) [file ppat.1012144.s003.tif]

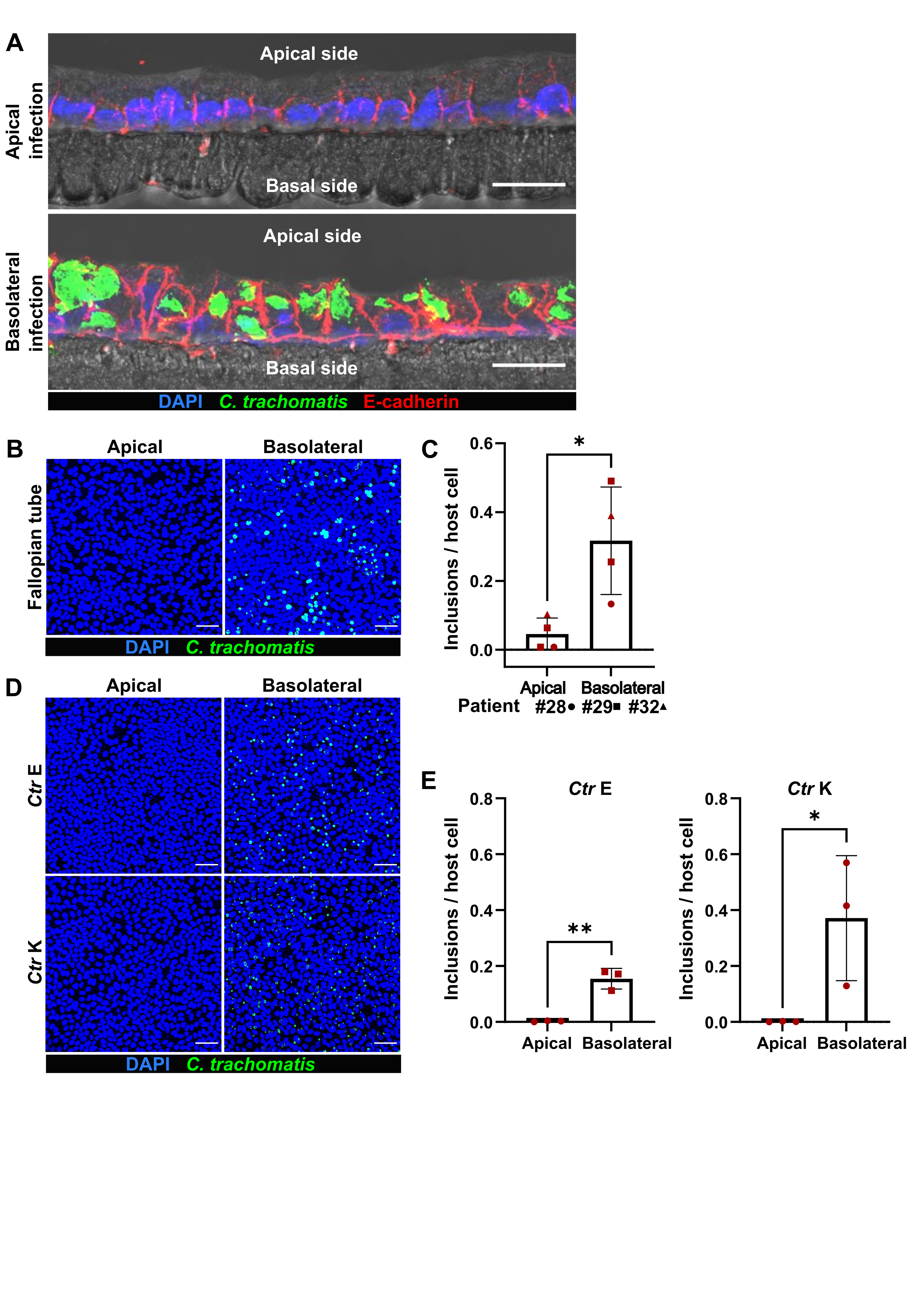

Supplement: S4 Fig — (A) Representative confocal microscopy image showing the results of apical (top image) and basolateral (bottom image) C. trachomatis infections in the cross-sections of polarized human primary gastric cells. Human primary gastric cells cultured in cell culture inserts were infected with C. trachomatis (MOI of 5) from the apical or basolateral surface and after 2 hours of incubation the inoculum was removed, and the cells were kept in fresh organoid medium. 24 hours p.i. the cells were washed and fixed with PFA. 5 μm histological cross-sections of the paraffin-embedded samples were used for the immunofluorescence analysis (blue: DAPI, green: C. trachomatis, red: E-cadherin). The fluorescence channels were merged with the bright field channel to visualize cell orientation. Scale bar: 20 μm. (B) Organoid-derived human primary fallopian tube cells grown on cell culture inserts were infected with GFP-expressing C. trachomatis (MOI of 5) from apical or basolateral surface․ After 2 hours of incubation, the inoculum was removed and the cells were kept in the fresh organoid medium. 24 hours p.i. the cells were fixed, stained and subjected to confocal microscopy. Shown are representative confocal fluorescence Z-stack images of four independent experiments (blue: DAPI, green: C. trachomatis). Scale bar: 50 μm. (C) The number of chlamydial inclusions and host cell nuclei in (B) was quantified in at least 4 fields of view per sample using Fiji. Data represent the mean ± SD from four independent experiments. Statistical analysis was performed by unpaired t-test (*P<0.05). The data point numbers and shapes below the graphs refer to the donor IDs used in the experiments. (D) Human primary gastric cells grown on cell culture inserts were infected with C. trachomatis serovar E (6.5 x 10^7^ chlamydial particles) and serovar K (5.5 x 10^7^ chlamydial particles) from the apical or basolateral surface and after 2 hours of incubation the inoculum was removed, and the cells were kept in fresh [file ppat.1012144.s004.tif]

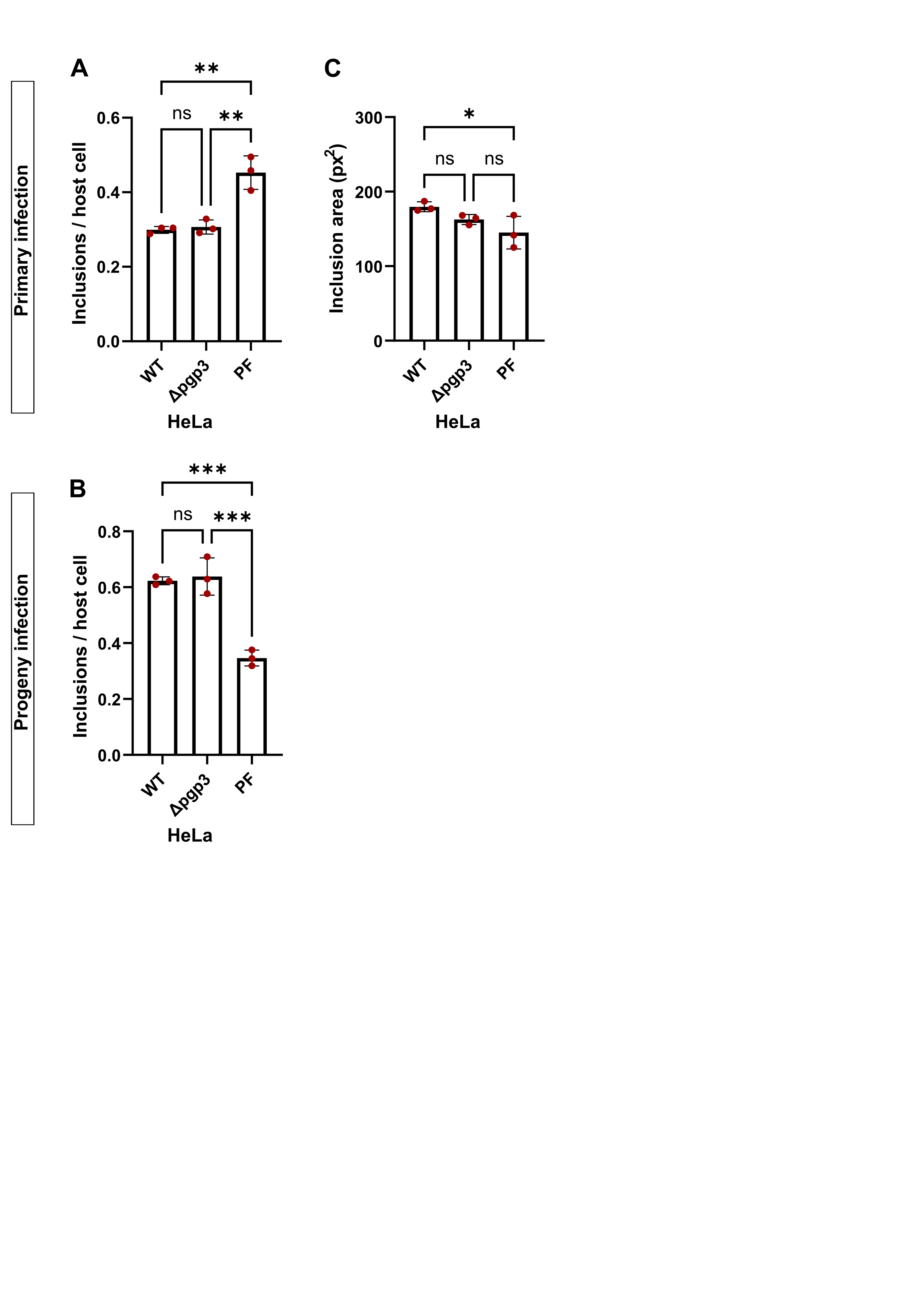

Supplement: S5 Fig — (A) Primary infection of HeLa cells infected with Ctr WT, Ctr Δpgp3 and Ctr PF. Subconfluent monolayers of HeLa cells were infected with the chlamydial derivatives at MOI of 0.5. 24 hours p.i. the cells were fixed, stained and the infection rate was determined by quantifying the number of inclusions and host cell nuclei in 14 fields of view per sample by automated microscopy. To assess the infectivity of the chlamydial progeny (B), the infected HeLa cells were lysed 48 hours p.i. and freshly seeded HeLa cells were infected with an aliquot of the lysates. 24 hours p.i. the cells were fixed, stained and the infection rate was determined in 14 fields of view per sample by automated microscopy. (C) The average size of the inclusions during primary infection was determined by automated microscopy in 14 fields of view per sample. All graphs represent the mean ± SD from three independent experiments. Statistical significance was determined by one-way ANOVA (ns = not significant, *P<0.05, **P<0.01, ***P<0.001). (TIF) [file ppat.1012144.s005.tif]
