# Supplementary material for: Infection of human organoids supports an intestinal niche for Chlamydia trachomatis
Source: PLoS Pathog. 2024 Aug 22;20(8):e1012144. doi: 10.1371/journal.ppat.1012144 (PMC11340892; doi:10.1371/journal.ppat.1012144)
Supplement: S1 Table — CM: conditioned medium; EGF: epidermal growth factor; FGF-10: fibroblast growth factor-10; TGF-β: transforming growth factor-β; IGF-1: insulin-like growth factor I; FGF-2: fibroblast growth factor-basic. (DOCX) [file ppat.1012144.s006.docx]

**S1 Table**. Media composition for human gastric, intestinal and fallopian tube organoids (CM: conditioned medium; EGF: epidermal growth factor; FGF-10: fibroblast growth factor-10; TGF-β: transforming growth factor-β; IGF-1: insulin-like growth factor I; FGF-2: fibroblast growth factor-basic)

| **Human gastric organoid medium** | | | |
| --- | --- | --- | --- |
| **Reagent** | **Supplier** | **Catalog Number** | **Final concentration** |
| Basal medium |  |  | 30% |
| WNT CM | stable cell line |  | 50% |
| R-Spondin CM | stable cell line |  | 10% |
| Noggin CM | stable cell line |  | 10% |
| B27 supplement | Thermo Fisher Scientific | 12587010 | 1x |
| N-acetylcysteine | Sigma Aldrich | A9165 | 1.25 mM |
| EGF | Peprotech | AF-100-15 | 50 ng/mL |
| FGF-10 | Peprotech | 100-26 | 100 ng/mL |
| Gastrin-I | Tocris | 3006 | 1 nM |
| TGF-β inhibitor | Tocris | 2939 | 2 uM |
| **Human intestinal organoid medium** | | | |
| **Reagent** | **Supplier** | **Catalog Number** | **Final concentration** |
| Basal medium |  |  | 30% |
| WNT CM | stable cell line |  | 50% |
| R-Spondin CM | stable cell line |  | 10% |
| Noggin CM | stable cell line |  | 10% |
| B27 supplement | Thermo Fisher Scientific | 12587010 | 1x |
| N-Ac | Sigma Aldrich | A9165 | 1.25 mM |
| EGF | Peprotech | AF-100-15 | 50 ng/mL |
| IGF-1 | Biolegend | 590904 | 100 ng/mL |
| FGF-2 | Peprotech | AF-100-18B | 50 ng/mL |
| Gastrin-I | Tocris | 3006 | 10 nM |
| TGF-β inhibitor | Tocris | 2939 | 0.5 uM |
| **Human fallopian tube organoid medium** | | | |
| **Reagent** | **Supplier** | **Catalog Number** | **Final concentration** |
| Basal medium |  |  | 40% |
| WNT CM | stable cell line |  | 25% |
| R-Spondin CM | stable cell line |  | 25% |
| Noggin CM | stable cell line |  | 10% |
| B27 supplement | Thermo Fisher Scientific | 17504044 | 1x |
| N2 supplement | Thermo Fisher Scientific | 17502048 | 1x |
| EGF | Peprotech | AF-100-15 | 50 ng/mL |
| FGF-10 | Peprotech | 100-26 | 100 ng/mL |
| Nicotinamide | Sigma | 3376 | 1 mM |
| TGF-β inhibitor | Tocris | 2939 | 0.5 uM |
